# Rising to the challenge: medical students as Doctors’ Assistants; an evaluation of a new clinical role

**DOI:** 10.30476/jamp.2020.87764.1320

**Published:** 2021-01

**Authors:** DANIELLE M LAVENDER, ANDREW P DEKKER, AMOL A TAMBE

**Affiliations:** 1 Department of Trauma and Orthopaedic Surgery, University Hospitals of Derby and Burton NHS Foundation Trust, UK; 2 University of Nottingham, UK

**Keywords:** Medical students, COVID-19, Doctors' assistants, Medical education, Pandemic

## Abstract

**Introduction:**

The COVID-19 Pandemic brought clinical placements to a halt for many UK medical students. A University Hospitals Trust offered clinical phase students the opportunity to support the National Health Service (NHS) in newly defined roles as Doctors’ Assistants (DAs). This study evaluates the experience of students working in a single NHS Trust. To our knowledge, this is the first report of medical students’ perspectives on taking up a novel clinical role in the UK.

**Methods:**

An anonymised novel electronic survey was sent to all 40 DAs across a single University Hospitals Trust via email to determine student perceptions of several aspects of the role, including its value to learning and development, impact on well-being, and benefit to the clinical environment. A formal statistical analysis was not required.

**Results:**

Of the total cohort participating in the programme, 32 DAs responded (80% response rate). The experience was considered valuable to multiple aspects of learning and development, particularly familiarisation with the role of a Foundation doctor. Levels of confidence in training and support were high, and most DAs felt valued as part of the clinical team, and experienced no mental health issues resulting from their role. 53% of the participants felt their work was necessary or valuable to the team, and all reported a positive experience overall.

**Conclusion:**

A new role allowed medical students to effectively provide clinical assistance during the COVID-19 pandemic. This provided immediate support to clinical teams as well as learning opportunities for the participants without detriment to their mental well-being, and could be a model for effective retention of medical students in clinical environments in the face of resurgence of COVID-19.

## Introduction

On the 30th January 2020, NHS England announced a Level 4 National Major Incident in response to severe acute respiratory syndrome coronavirus 2, also known as coronavirus disease 2019 (COVID-19) ( 1 ). The World Health Organisation declared the outbreak a pandemic on 11th March 2020 ( 2 ), and as the extent of the impact in the United Kingdom (UK) became clearer, medical schools reacted by suspending clinical placements for students. Final year students graduated early, as part of an initiative to allow them to start work as Foundation doctors. Although this was not appropriate for students in earlier years, it was understood that many students would wish to use their skills to support the NHS ( 3 ). The Medical Schools Council released a statement on the 24th March 2020 aimed at medical schools, NHS Trusts and students, containing guidelines relating to medical student volunteers in the NHS ( 4 ). An 8-week programme for senior medical students to provide assistance on the wards in a new role, known as Doctors’ Assistant (DA) was rolled out at our hospital trust. 

The Doctors’ Assistant job description was provided to participants via email. It outlined the purpose of the role: supporting doctors, assisting with ward and administrative activities and therefore relieving some of the demands on juniors in particular. An induction was provided to the participants before they were assigned to various wards or clinical areas across the hospital. 

Evaluating new initiatives allows application of learning points to improve future programmes. Thus far, there is evidence that a large proportion of medical students are willing to assist or continue placements in clinical areas during the pandemic ( 5 , 6 ). One study in Germany suggested that pre-final year student volunteers felt helpful and some acquired new skills, though only 5 of the respondents were in a hospital ( 5 ). However, to our knowledge there has been no investigation into roles tailored to the competencies and expectations of medical students. Insight into the areas of maximum value in clinical assignments may be useful given the ongoing restriction to clinical placements, and the potential need for adaptations and reduction in patient contact for medical students, imposed upon us by the COVID pandemic. 

This study aimed to determine student perceptions on several aspects of the DA programme, including the value to learning and development, impact on wellbeing, induction and preparation, benefit to the hospital wards, and supervision and support. 

## Methods

**Type of Study:** An anonymised electronic survey created with Google Forms was used to collect students’ opinions and experiences as Doctors’ Assistants (DAs). The survey was constructed in conjunction with the questionnaire used in ( 7 ). Discussions with DA participants highlighted several common themes that were used in formulating the questions. 

**Sampling:** The survey was issued via email to all 40 DAs across a single University Hospitals Trust and was open between 22nd May and 24th June 2020. DAs had been in clinical roles for 5 weeks at the launch of the survey. 

**Tools:** DAs were asked to provide anonymised, confidential information on aspects of the programme. Emotion and opinion can be appropriately investigated using surveys ( 7 , 8 ), and DAs were asked for their views on their induction, support, learning and development, value on the ward, concerns, and mental health and well-being. Factual information was also gathered, including the number of hours worked, the area(s) to which DAs were assigned, and what activities they undertook. 

The questions were a mixture of formats, including free text, multiple choice, dichotomous and Likert scale responses. 

Values for discrete responses are presented with percentages. Free text responses were analysed by the lead author (DML) to inform the results and conclusions. Formal statistical analysis was not required.

Ethical permission was not deemed necessary as this study represents a service evaluation of opinions using a voluntary and anonymised survey format with no impact on patient treatment. Permission to circulate the survey was obtained from the appropriate authority.

## Results

From a total of 40 DAs in the Trust, 32 responded to the survey, giving a response rate of 80%. 

**Figure 1:**
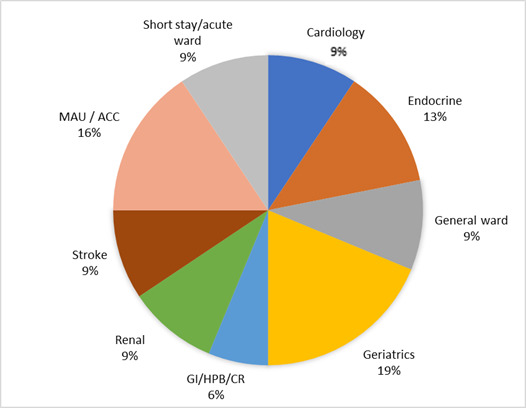
Pie chart of Doctors' Assistants (DAs) deployment area.

DAs were deployed across a range of specialties, as shown in Figure 1. All DAs worked 20 hours a week. A three-day week was the most common pattern, with the majority finding this reasonable and convenient.

MAU: Medical Assessment Unit, ACC: Ambulatory Care Centre, GI: Gastrointestinal medicine, HPB: Hepato-Pancreatico-Biliary services, CR: Colorectal surgery.

### Induction and preparation

As shown in Table 1, almost all DAs (91%) agreed or strongly agreed that they were given appropriate training prior to being deployed and 88% were confident in their understanding of the appropriate Personal Protective Equipment (PPE) for each clinical situation. After induction, the majority (63%) felt clear on what their role would involve, with only 3% disagreement noted. 

**Table 1:** Extent to which Doctors' Assistants (DAs) agreed with various statements regarding their induction.

Statement	Strongly disagree	Disagree	Neutral	Agree	Strongly agree
I felt confident I knew what the appropriate Personal Protective Equipment (PPE) was for each clinical situation.	0%	3%	9%	28%	59%
Prior to deployment, I received appropriate clinical skills/other training.	0%	3%	6%	56%	34%
After induction, I felt clear about what my role would involve.	0%	3%	34%	44%	19%

### Support and Value

**Figure 2:**
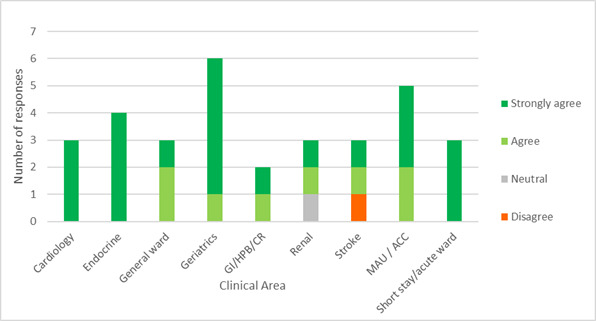
Chart showing the Doctors' Assistants (DAs) levels of agreement with the statement “I felt well supported in my clinical role.” by area, from strongly disagree to strongly agree.

The overwhelming majority of DAs (94%) felt well supported in their clinical roles, (Figure 2). Direct supervision was provided by doctors at all levels, as well as nursing or other healthcare professionals, and many DAs were supervised by multiple colleagues. Foundation doctors were involved in the supervision of 78% of respondents. Direct consultant level supervision was reported by 31%. 

MAU: Medical Assessment Unit, ACC: Ambulatory Care Centre, GI: Gastrointestinal medicine, HPB.

**Figure 3:**
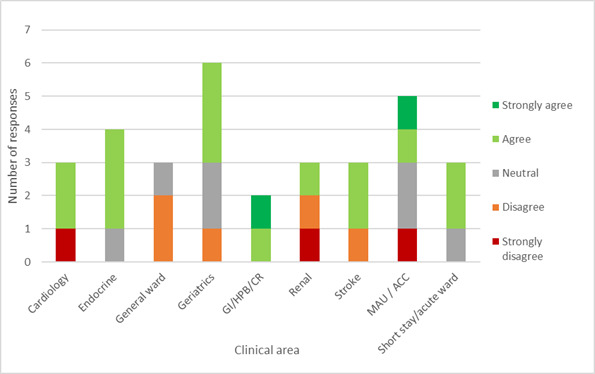
Perceived value to the ward by area. Extent to which Doctors' Assistants (DAs) agreed with the statement “I felt I was providing a necessary/ valuable contribution to my ward or clinical area".

Overall, 78% of DAs agreed or strongly agreed they felt valued as a member of the clinical team; however, this agreement was not shared by 22% (Table 2). Just over half of the respondents (53%) agreed or strongly agreed that their contributions were valuable/ necessary, with 25% disagreement (Table 2, Figure 3).

**Table 2:** Extent to which Doctors' Assistants (DAs) agreed with various statements regarding their value

Statement	Strongly disagree	Disagree	Neutral	Agree	Strongly agree
I felt valued as a member of the clinical team.	0%	16%	6%	47%	31%
I felt I was providing a necessary/valuable contribution to my ward or clinical area.	9%	16%	22%	47%	6%

MAU: Medical Assessment Unit, ACC: Ambulatory Care Centre, GI: Gastrointestinal medicine, HPB: Hepato-Pancreatico-Biliary services, CR: Colorectal surgery.

DAs were asked to recount any concerns they had prior to starting the role, and comment on whether these materialised. Being asked to work outside competence was reported by several DAs as being a concern prior to their deployment, but one that did not actually materialise in practice. Another common theme was apprehension over not being ‘useful’ on the ward or being a hindrance; this did transpire for several DAs. Some uncertainty over what the role would entail was also reported, as well as concern over whether DAs would be well received and integrated into the clinical team. Actual experiences were mixed in these respects. 

Minimal concerns were raised regarding personal protective equipment (PPE) or personal risk, and there were no reported issues regarding the availability of PPE. 

### Learning

**Figure 4:**
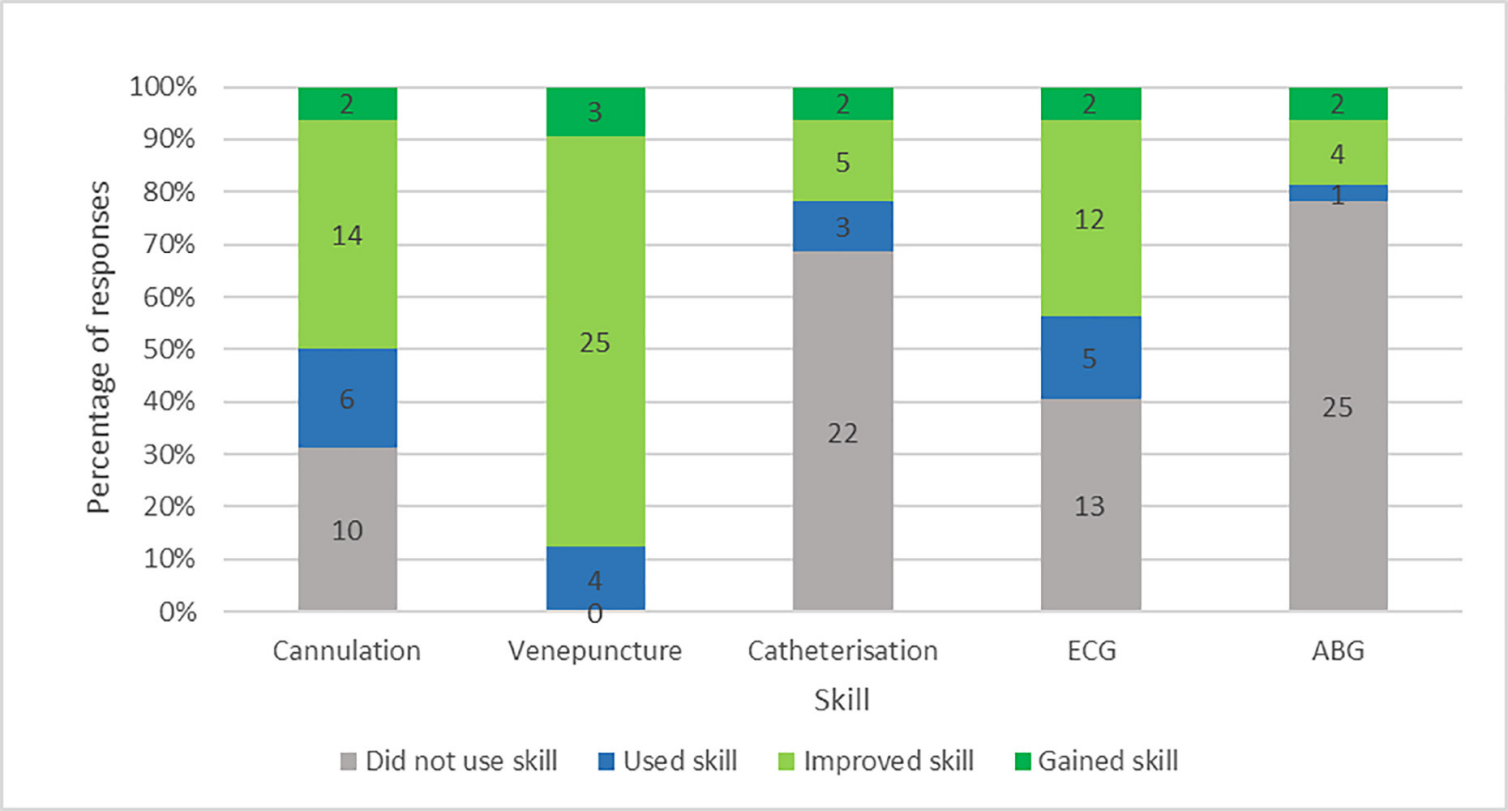
The use of clinical skills in Doctors' Assistants (DAs) placements.

The majority of DAs (91%) reported improving and/or gaining one or more of the listed clinical skills as part of their placement, and every DA used at least one clinical skill. Figure 4 shows the skills and their use in more detail. Additional skills reported included cognitive assessments, and initial assessment of the acute patient. 

ECG: Electrocardiogram. ABG: Arterial Blood Gas. Numbers show absolute values of responses.

**Figure 5:**
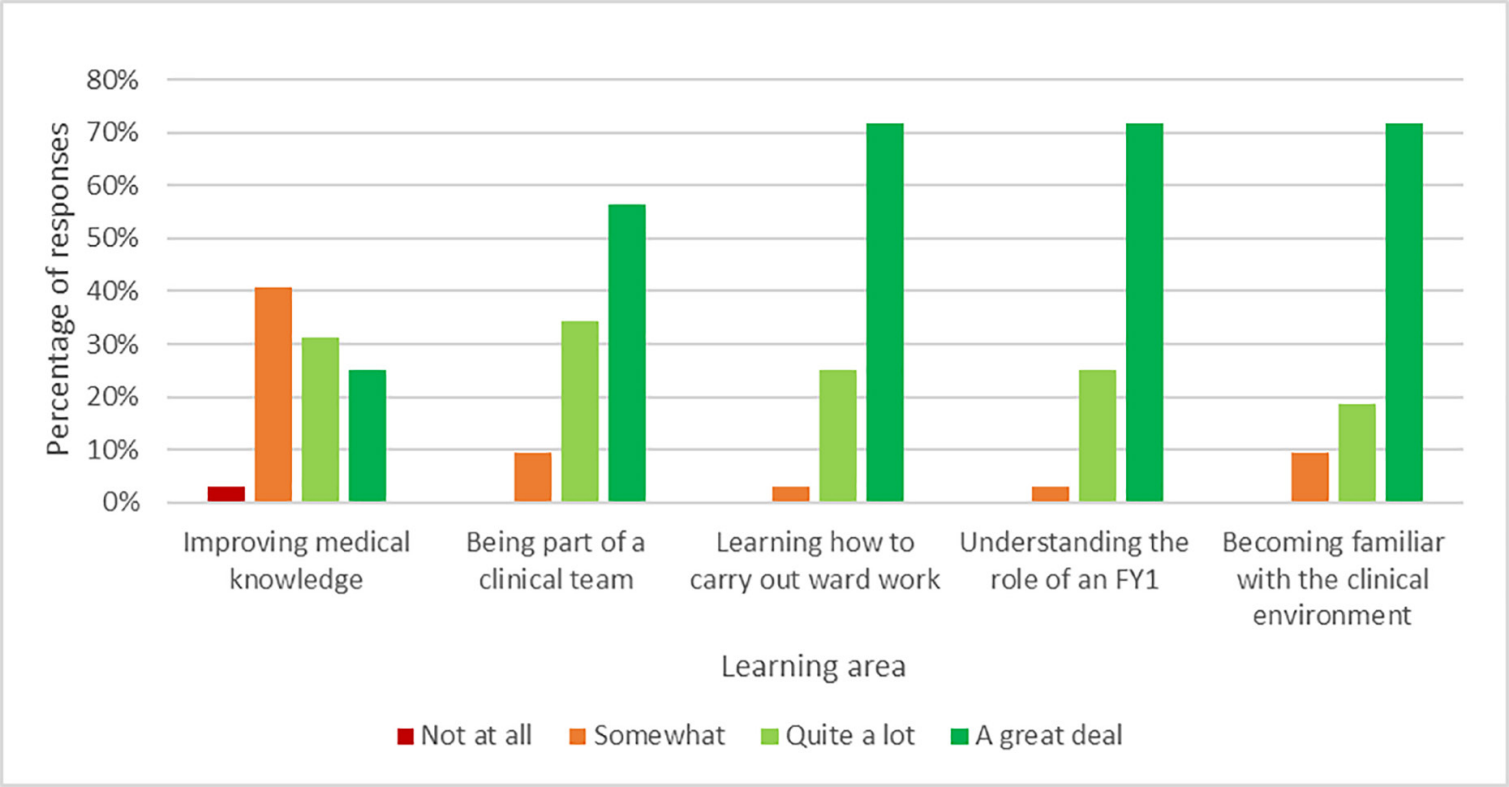
Doctors' Assistants (DAs) assessments of the value of the placement to aspects of learning.

Figure 5 suggests the vast majority of DAs derived “a great deal” of benefit from the programme in becoming familiar with clinical teams (56%), environments (72%), ward work (72%) and Foundation Year 1 (FY1) roles (72%). All DAs reported value to their learning in these areas. Placements were not as highly rated for improving medical knowledge, although still only a small minority (3%) suggested it had been of no benefit at all in this respect. 

FY1: Foundation Year 1 doctor.

Ward-based teaching was reported by 78% of DAs. Most DAs (84%) continued with some form of study over the placement period. 

All DAs felt the placement made them more prepared to commence work as a newly qualified junior doctor, with 69% feeling that it had helped “a great deal”, 28% feeling it helped “quite a lot” and the remaining 3% believing it had helped “moderately” in this respect. A majority of comments highlighted the value of the program in increasing knowledge and competence in the administrative aspects of a junior doctor’s role, such as discharge summaries and ward round documentation. 

### Mental health and well-being

The survey requested information on mental health and wellbeing relating to the DA role. Overall, 91% of respondents experienced no difficulties, and some comments expressed the view that the activity and purpose provided by the scheme had been beneficial to well-being. The few mental health concerns raised included anxiety (6%), and stress relating to illness (3%). 

### Overall

**Figure 6:**
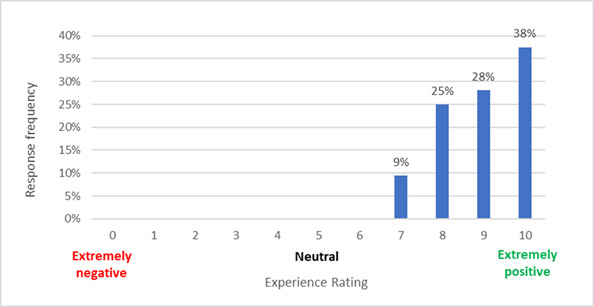
Doctors' Assistants (DAs) overall rating of their experience, where 0 is extremely negative, and 10 is extremely positive

All respondents to the survey reported a positive experience overall (Figure 6). On a scale of 0 to 10, the mean score attributed to the experience was 9 (0dp).

## Discussion

This study aimed to evaluate the DA programme from a medical student perspective, considering the benefit DAs both derived and provided in the clinical context. 

The survey had a good response rate (80%) and most DAs reported a very positive overall experience with a clear benefit to individual learning and development. The participants overwhelmingly felt the experience helped prepare them for commencing work as a newly qualified junior doctor. A recent nationwide survey of final year medical students found that the disruption of the “student assistantship” was the only factor that had a statistically significant impact on students’ confidence to start assisting in hospitals earlier than anticipated ( 9 ). The DA programme may therefore convey a huge potential advantage in having a confident and ‘hospital-aware’ cohort of students available should this need arise. Most DAs received some form of teaching on the ward, suggesting firstly that receiving teaching and providing a useful clinical service were not mutually exclusive, and secondly that clinicians were generally able to provide some form of education, even during an unprecedented healthcare crisis. This underlines the utility of this programme in providing continuing medical education should there be any further interruption to the formal medical school course due to rising COVID-19 cases. 

In response to a question regarding top learning points from the programme, multiple survey participants reported experiencing and learning about collaborative and multidisciplinary team working, maintaining good clinical notes, effective admission/discharge procedures, understanding effective handovers and gaining confidence in basic clinical skills. These are all important attributes that underpin the making of a good doctor.

The considerable prevalence of mental health problems in healthcare workers during the pandemic reported in other studies ( 7 , 10 ) was not reflected in the DA cohort. Hypotheses that clinical positions for medical students might have a high risk of psychological consequences such as PTSD ( 11 ) do not appear to be borne out by this survey, and several DAs suggested the role had been protective to mental well-being. The remarkably high proportion experiencing no issues (91%) could be attributed to the good level of support, appropriate training and manageable hours outweighing any stresses. Another factor may have been the suitable level of responsibility of the role; no DA felt pressure to work outside their competence. These findings provide some reassurance that DA programmes can be introduced or restarted without major adverse consequences to student mental health. 

Given that participation in the programme was entirely voluntary, the high satisfaction and low incidence of problems with well-being may also partly reflect the self -selection of those students more positively predisposed towards the programme, or more likely to be able to derive a net benefit. 

Inductions for Doctors’ Assistant roles began 3 weeks after the imposition of the national lockdown ( 12 ), and sometime after the outbreak began to impact practices at the hospital Trust. This meant DAs entered the clinical environment at a time when PPE guidance was relatively stable, and may explain the high level of confidence in PPE use amongst the cohort, particularly when compared to doctors in training, who experienced daily PPE guidance updates ( 7 ). 

Additional personnel in the clinical environment potentially pose an increased infection risk to staff, patients, and the wider community. At the time of the survey, these risks, combined with the increased teaching burden students place on clinical colleagues, were considered too great to justify the presence of medical students in hospitals for educational purposes alone ( 13 ), hence the suspension of usual placements. The presence of medical students can be justified where the potential value of their contribution to the clinical environment outweighs the additional risk they pose. DA roles represent unchanged clinical risks, but altered benefits, in aiming to help meet a clinical need created by the increased demands on the NHS, or for that matter any health care system, due to COVID-19. As such, the programme could potentially be advocated as a model for bringing students into the clinical environment in a justifiable way. 

DAs potentially benefit wards by reducing the workload for junior doctors, enabling greater rota compliance, and relieving doctors for redeployments elsewhere. Though a reasonable control study model is not available to substantiate these claims, over half the DAs felt their contribution was necessary or valuable to the clinical environment (53%). Even had this not been the case, it could be argued that the unpredictable nature of the pandemic alone would have justified the programme as a necessary move to create reserve capability within the hospital. Additionally, the professional development experienced by all DAs, if not immediately advantageous, has the potential to benefit the Trust in the event of reinstatement of the roles. By implementing this programme, the Trust not only gained immediate assistance and reserve capacity, but created a trained group of students likely to be more effective in any future deployments. 

Whilst the proportion of DAs receiving direct consultant supervision was small, almost all agreed that they were well supported in their role. It is therefore reasonable to conclude that DAs generally did not require significant direct consultant supervision at all times, suggesting a minimal ongoing burden for consultants on wards with DAs. 

The small cohort makes it difficult to identify those areas where DAs were most helpful. While many of the concerns the participants had prior to joining the programme did not largely materialise, a small number of DAs took some time to integrate within their teams and find a defined role. There were several comments relating to usefulness, availability of work, and staffing levels, suggesting that the programme could have been improved by targeting placements to those areas where students could be more effective. This may be an important factor to consider in future schemes, perhaps by having a flexible allocation system to respond to fluctuations in clinical need. 

### Limitations

Potential limitations to this evaluation include the relatively small numbers (despite the high response rate), representing responses from a single University Hospital Trust, with the majority of participants from one UK medical school. DAs were volunteers, and may therefore not be representative of a cross-section of medical students. They may be more inclined to value the experience than others who did not apply. 

Due to the unprecedented pandemic situation, and the time-limited nature of the programme, it was not possible to formally validate the survey. However, the questions asked in this survey have intrinsic validity, and were developed with first-hand insight from the programme. 

Over the pandemic period, there have been other publications relating to surveys ( 5 - 7 , 9 ) that are not described as validated.

## Conclusion

To our knowledge, this is the first survey analysis of medical student perceptions of a new clinical role. Since planned placements for medical students were suspended, this study provides an insight into students in hospital environments impacted by COVID-19 as Doctors’ Assistants. 

It demonstrates how the medical students rose to the challenge to effectively provide clinical assistance during the pandemic. This offered immediate support to clinical teams across a spectrum of activities from administrative to minor procedures, and a degree of contingency should demands on the hospital escalate. The programme allowed participants to gain valuable experience in a hospital environment, and could be an effective model for the retention of medical students in clinical environments, in the face of a resurgence of the virus. 
